# Sudden Cardiac Arrest in Proximal Femur Fracture: The Role of Admission Blood Parameters

**DOI:** 10.7759/cureus.103983

**Published:** 2026-02-20

**Authors:** Hüseyin Aldemir

**Affiliations:** 1 Emergency Medicine, Afyonkarahisar State Hospital, Afyonkarahisar, TUR

**Keywords:** fat embolism syndrome (fes), geriatric trauma, massive pulmonary embolism, neutrophil-to-lymphocyte ratio (nlr), proximal femur fracture, sudden cardiac arrest

## Abstract

Proximal femur fractures in the elderly are associated with high mortality rates due to reduced physiological reserve and a high risk of thromboembolic events, where admission laboratory parameters can serve as early indicators of clinical vulnerability. This case highlights a catastrophic clinical deterioration in a 92-year-old man who was admitted following a fall resulting in a displaced right intertrochanteric fracture. On admission, the patient was hemodynamically stable, but laboratory findings revealed an elevated neutrophil-to-lymphocyte ratio (NLR) of 10.1 and significant hyponatremia (126 mmol/L), reflecting high physiological stress and reduced compensatory reserve. Approximately 10 hours after admission, during preoperative preparation, he developed sudden dyspnea, hypotension, and pulseless electrical activity (PEA). Despite 30 minutes of advanced cardiac life support, the patient died. Arterial blood gas analysis during resuscitation showed profound hypoxemia (PaO₂ 42 mmHg) and severe metabolic acidosis (pH 6.98). Early mortality in geriatric hip fractures can occur even before surgical intervention, and abnormal admission parameters like elevated NLR and electrolyte imbalances should alert clinicians to a reduced compensatory reserve. In cases of sudden PEA arrest in the emergency department (ED), both massive pulmonary embolism and early-onset fat embolism syndrome should be considered in the differential diagnosis as plausible but unconfirmed causes.

## Introduction

Proximal femur fractures in elderly patients represent a major public health challenge, frequently associated with significant early mortality and a high burden of comorbidities [1,2]. These injuries often trigger a systemic inflammatory response, leading to life-threatening thromboembolic and cardiopulmonary complications such as massive pulmonary embolism (PE) and fat embolism syndrome (FES) [3,4]. Studies have shown that the risk of fatal PE is particularly high within the first 24-48 hours of injury, often occurring before surgical stabilization can be achieved [4]. Furthermore, admission laboratory markers, including the neutrophil-to-lymphocyte ratio (NLR) and serum sodium levels, may reflect the patient's underlying physiological stress and reduced compensatory reserve, which are recognized as predictors of mortality in geriatric trauma patients [5,6]. Identifying these high-risk patients upon emergency department (ED) admission is vital, as sudden clinical deterioration may lead to a diagnostic challenge, especially when symptoms of PE and FES overlap [1-3].

## Case presentation

**Figure 1:**
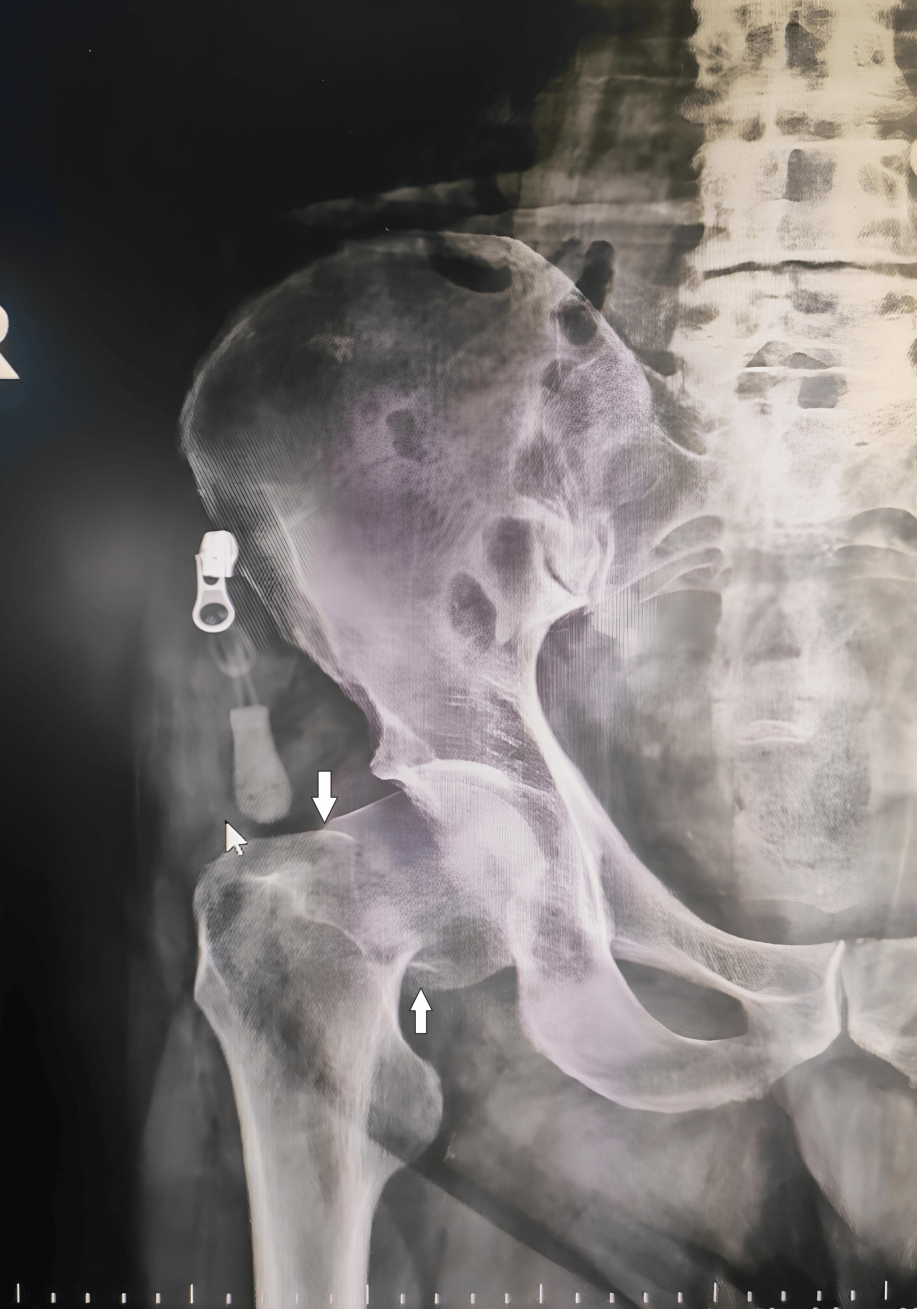
Anteroposterior pelvic radiograph showing a displaced right intertrochanteric fracture

A 92-year-old male patient was brought to the ED after a fall from standing height at home. He had been fully ambulatory prior to the fall. There was no known history of cardiovascular disease, malignancy, anticoagulant use, or recent immobilization. On admission, the patient was conscious, cooperative, and oriented. Vital signs were stable with a blood pressure of 140/85 mmHg, heart rate of 90 beats/min, respiratory rate of 18 breaths/min, oxygen saturation of 96% on room air, and body temperature of 36.6°C. Electrocardiography showed a normal sinus rhythm. Physical examination revealed pain, shortening, and external rotation of the right lower extremity. Pelvic radiography demonstrated a displaced right intertrochanteric fracture (Figure 1).

Initial laboratory evaluation at ED admission revealed several abnormal blood parameters (Table 1).

**Table 1:** Admission laboratory findings and reference ranges

Laboratory parameter	Admission value	Reference range
Hemoglobin	9.6 g/dL	13.5-17.5 g/dL
White blood cell count	13.9 × 10⁹/L	4.5-11.0 × 10⁹/L
Neutrophil count	11.1 × 10⁹/L	2.0-7.0 × 10⁹/L
Lymphocyte count	1.1 × 10⁹/L	1.0-3.0 × 10⁹/L
Neutrophil-to-lymphocyte ratio (NLR)	10.1	1.0-3.0
Serum sodium	126 mmol/L	135-145 mmol/L
Serum creatinine	1.4 mg/dL	0.7-1.3 mg/dL
Blood urea nitrogen (BUN)	42 mg/dL	7-20 mg/dL
C-reactive protein (CRP)	19.4 mg/L	<5 mg/L

During the preoperative evaluation and clinical preparation process, the patient remained under observation in the ED, while preoperative anesthesia evaluation and surgical preparation were initiated.

Approximately 10 hours after admission, during preoperative preparation, the patient suddenly developed acute dyspnea and hypotension, followed rapidly by loss of consciousness. The cardiac monitor revealed pulseless electrical activity (PEA). Immediate cardiopulmonary resuscitation was initiated according to advanced cardiac life support protocols. Despite 30 minutes of resuscitative efforts, return of spontaneous circulation could not be achieved. In contrast to the initial arterial blood gas analysis, the analysis obtained during cardiac arrest demonstrated severe hypoxemia, hypercapnia, and profound metabolic acidosis accompanied by markedly elevated lactate levels, consistent with severe tissue hypoperfusion (Table 2).

**Table 2:** Comparison of arterial blood gas (ABG) values and reference ranges

Arterial blood gas analysis parameter	Admission (stable)	During cardiac arrest (PEA)	Reference range
pH	7.38	6.98	7.35-7.45
PaO₂	86 mmHg	42 mmHg	80-100 mmHg
PaCO₂	40 mmHg	58 mmHg	35-45 mmHg
HCO₃⁻	24 mmol/L	13 mmol/L	22-26 mmol/L
Lactate	1.9 mmol/L	11.8 mmol/L	0.5-2.2 mmol/L

## Discussion

Early mortality following proximal femur fractures remains a significant clinical concern, particularly in elderly patients [1,2]. Delays in surgical management and early physiological derangements have been associated with adverse short-term outcomes after hip fractures [2-4]. Admission laboratory abnormalities may help identify patients with limited physiological reserve in this vulnerable population [5,6]. Specifically, elevated NLR and electrolyte imbalances, such as hyponatremia, as seen in our patient, should be viewed as risk indicators of clinical frailty rather than direct predictors of acute embolic events [1,7]. In the present case, the patient demonstrated multiple abnormal laboratory findings at ED admission despite being hemodynamically stable and fully conscious. The sudden clinical deterioration characterized by acute dyspnea and PEA suggests an acute obstructive cause of cardiac arrest rather than a primary cardiac etiology. Massive PE was considered a potential clinical hypothesis among other differential diagnoses, given the abrupt onset of respiratory compromise, rapid hemodynamic collapse, severe hypoxemia, and refractory PEA arrest. Elderly patients with lower extremity fractures may be at increased risk of thromboembolic complications, even in the early post-injury period [4]. FES was also considered in the differential diagnosis. Although FES is more commonly reported 12-72 hours after injury, early presentations have been described, particularly in elderly patients with long bone or proximal femur fractures [3]. Although the classic triad was not fully present, FES cannot be definitively excluded. Fulminant forms of FES have been described where rapid cardiac arrest occurs without petechial rash or neurological findings. Therefore, a more balanced comparison must be made, as both massive PE and early-onset FES are plausible causes for such a catastrophic collapse [3,4]. This case highlights the diagnostic challenges faced in the ED when catastrophic deterioration occurs before definitive imaging can be obtained. In the absence of confirmatory evidence, such as D-dimer levels, CT pulmonary angiography, bedside echocardiography, or a post-mortem examination, the diagnosis of massive PE or FES remains speculative and unconfirmed.

## Conclusions

In conclusion, proximal femur fractures in geriatric patients carry a significant risk of sudden, catastrophic complications that may occur even before surgical intervention. While admission laboratory parameters, specifically an elevated NLR and hyponatremia, are associated with increased mortality, they should be interpreted as risk indicators reflecting a compromised physiological reserve rather than direct predictors of acute embolic events. In cases of sudden cardiovascular collapse, massive PE and FES represent a formidable diagnostic challenge. However, in the absence of confirmatory imaging, D-dimer levels, or autopsy findings, the definitive etiology in this case remains a speculative and unconfirmed diagnosis. This report underscores the necessity of maintaining a high clinical suspicion for both PE and FES in the emergency department, as these events can lead to rapid clinical deterioration in patients with limited compensatory mechanisms, even when a definitive diagnostic conclusion cannot be reached.
